# Effect of the Osteotomy Inclination Angle in the Sagittal Plane on the Posterior Tibial Slope of the Tibiofemoral Joint in Medial Open-Wedge High Tibial Osteotomy: Three-Dimensional Computed Tomography Analysis

**DOI:** 10.3390/jcm10184272

**Published:** 2021-09-21

**Authors:** Jai Hyun Chung, Chong Hyuk Choi, Sung-Hwan Kim, Sung-Jae Kim, Seung-Kyu Lee, Min Jung

**Affiliations:** 1 Department of Medicine, Yonsei University Graduate School, Seoul 03722, Korea; jjhsky0843@gmail.com; 2 Arthroscopy and Joint Research Institute, Department of Orthopaedic Surgery, Yonsei University College of Medicine, Seoul 03722, Korea; choi8422@yuhs.ac (C.H.C.); orthohwan@yuhs.ac (S.-H.K.); severanscopy@yuhs.ac (S.-J.K.); 3 Department of Orthopaedic Surgery, Yonsei University College of Medicine, Seoul 03722, Korea; terry1376@yuhs.ac

**Keywords:** osteoarthritis, high tibial osteotomy, posterior tibial slope, inclination angle

## Abstract

The posterior tibial slope of the tibiofemoral joint changes after medial open wedge high tibial osteotomy (MOWHTO), but little is known about the effect of the sagittal osteotomy inclination angle on the change in the posterior tibial slope of the tibiofemoral joint. The purpose of this study was to investigate the effect of the osteotomy inclination angle in the sagittal plane on changes in the posterior tibial slope after MOWHTO by comparing how anterior and posterior inclination affect the posterior tibial slope of the tibiofemoral joint. The correlation between the osteotomy inclination angle and the postoperative posterior tibial slope angle was also assessed. Between May 2011 and November 2017, 80 patients with medial compartment osteoarthritis who underwent MOWHTO were included. The patients were divided into two groups according to the sagittal osteotomy inclination angle on the 3D reconstructed model. Patients with an osteotomy line inclined anteriorly to the medial tibial plateau line were classified into group A (58 patients). Patients with posteriorly inclined osteotomy line were classified as group P (22 patients). In the 3D reconstructed model, the preoperative and postoperative posterior tibial slope, osteotomy inclination angle relative to medial tibial plateau line in sagittal plane, and gap distance and ratio of the anterior and posterior osteotomy openings were measured. The preoperative and postoperative hip-knee-ankle angle, weight-bearing line ratio, and posterior tibial slope were also measured using plain radiographs. In the 3D reconstructed model, the postoperative posterior tibial slope significantly increased in group A (preoperative value = 9.7 ± 2.9°, postoperative value = 10.7 ± 3.0°, *p* < 0.001) and decreased in group P (preoperative value = 8.7 ± 2.7°, postoperative value = 7.7 ± 2.7°, *p* < 0.001). The postoperative posterior tibial slope (group A = 10.7 ± 3.0°, group P = 7.7 ± 2.7°, *p* < 0.001) and posterior tibial slope change before and after surgery (group A = 1.0 ± 0.8°, group P = −0.9 ± 0.8°, *p* < 0.001) also differed significantly between the groups. The Pearson correlation coefficient was 0.875 (*p* < 0.001) for the osteotomy inclination angle, and multivariate regression analysis showed that the only significant factor among the variables was the sagittal osteotomy inclination angle (β coefficient = 0.216, *p* < 0.001). The posterior tibial slope changed according to the osteotomy inclination angle in the sagittal plane after MOWHTO. The postoperative posterior tibial slope tended to increase when the osteotomy line was inclined anteriorly with respect to the medial tibial plateau line but decreased when the osteotomy line was inclined posteriorly. To avoid inadvertent change of posterior tibial slope, close attention needs to be paid to maintaining the sagittal osteotomy line parallel to the medial joint line during MOWHTO.

## 1. Introduction

Medial open wedge high tibial osteotomy (MOWHTO) is an established, effective operative treatment for relatively young and active patients with osteoarthritis in the medial compartment of the tibiofemoral joint and varus deformity [1,2]. MOWHTO reduces the pressure of the medial compartment in the tibiofemoral joint by realigning the mechanical axis from the medial to the lateral side in the coronal plane [3,4]. This procedure has been reported to have good long-term clinical outcomes such as reduced pain and improved knee joint function [5,6,7]. Previous studies on MOWHTO tended to focus mainly on the change of alignment in the coronal plane regarding correction angle and postoperative coronal realignment [8,9,10]. However, MOWHTO is the three-dimensional (3D) surgical procedure. Therefore, the postoperative effect of osteotomy on the proximal tibia should also be considered in the sagittal plane. In addition to correction in the coronal plane, maintaining alignment in the sagittal plane has been reported to be important. The change in the posterior tibial slope in the sagittal plane influences the biomechanics of the knee joint. The increase in posterior tibial slope of the tibiofemoral joint leads to anterior translation of the tibia relative to the femur, thus developing an overload on the anterior cruciate ligament [11,12]. An increased posterior tibial slope of the tibiofemoral joint could also cause a redistribution of pressure into the posterior tibia in the anterior cruciate ligament-deficient knee, resulting in degenerative change in articular cartilage [13] and an increase of vertical vector force onto the patellofemoral joint, which could lead to osteoarthritis of the patellofemoral joint [14,15].

Despite efforts to maintain the posterior tibial slope of the tibiofemoral joint after MOWHTO, the posterior tibial slope tends to change after MOWHTO [12,16]. Change in posterior tibial slope before and after MOWHTO can be affected by various factors, including the osteotomy opening gap ratio between the anterior and posterior cortex [17,18]. Recently, the inclination angle of osteotomy in the sagittal plane has also been reported to affect change in the posterior tibial slope [19]. However, to the best of our knowledge, there has been no comprehensive study on the effect of the osteotomy inclination angle in the sagittal plane on changes in the posterior tibial slope after MOWHTO, including the correlation between the osteotomy inclination angle and postoperative posterior tibial slope angle. Therefore, the purpose of this study was to investigate the effect of the osteotomy inclination angle in the sagittal plane on the change in posterior tibial slope after MOWHTO by comparing how anterior and posterior inclination affect the posterior tibial slope and assessing the correlation between the osteotomy inclination angle and postoperative posterior tibial slope angle. The measurement of values in this study was performed not only in two-dimensional radiographs, but also in 3D reconstructed images. It was hypothesized that anterior or posterior inclination of osteotomy in the sagittal plane would influence the change in the posterior tibial slope after MOWHTO. 

## 2. Materials and Methods

### 2.1. Study Participants

**Figure 1:**
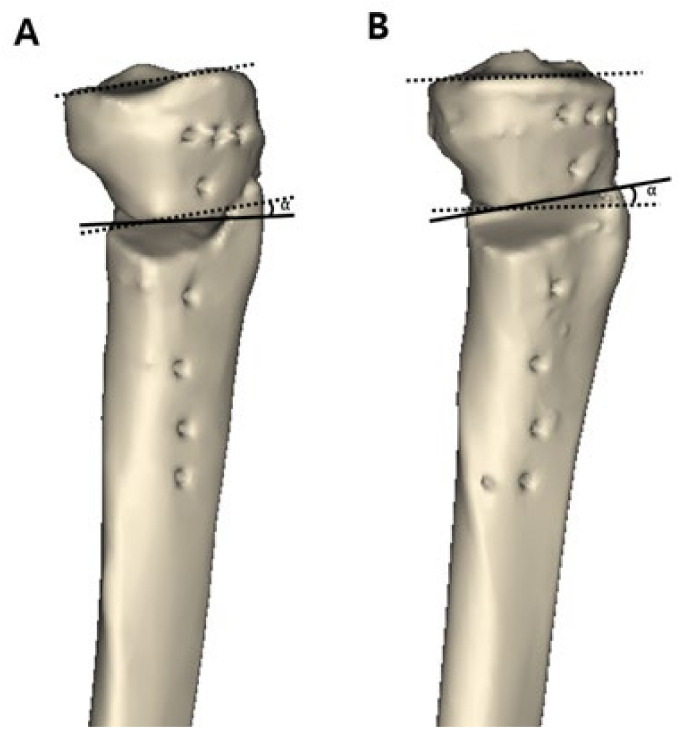
The patients were divided into two groups according to the osteotomy inclination angle in the sagittal plane in a three-dimensional reconstructed model. On the true lateral view, a black dotted line was drawn as a medial tibial plateau line. A black solid line was drawn as a sagittal osteotomy line on the anteromedial aspect of the lowermost part of the proximal tibial segment. The angle (α°) formed by these two lines was defined as the osteotomy inclination angle. (**A**) Group A, the osteotomy line is inclined anteriorly with respect to the medial tibial plateau line. (**B**) Group P, the osteotomy line is inclined posteriorly with respect to the medial tibial plateau line.

After approval by the institutional review board of our institution, patients with medial compartment osteoarthritis who underwent MOWHTO between May 2011 and November 2017 were retrospectively reviewed. A total of 169 knees were treated with MOWHTO during the study period. Patients who met the following criteria, including the surgical indications of MOWHTO, were included in the study: (1) symptomatic medial compartment osteoarthritis with Kellgren-Lawrence grade III or more, (2) varus deformity of the lower extremity >5°, (3) preserved cartilage in the lateral compartment (International Cartilage Repair Society [ICRS] grade [20] I or II), and (4) age of <65 years. Patients with the following criteria were excluded from the study: (1) patients who did not have a postoperative computed tomography (CT) scan for the full length of the tibia and reconstructed 3D model; (2) a history of previous surgery on the affected knee; and (3) the presence of ligament injury on the affected knee. After applying the inclusion and exclusion criteria, 80 knees were included in this study. The patients were divided into two groups according to the osteotomy inclination angle in the sagittal plane in the 3D model reconstructed CT scan images. When the osteotomy line was inclined anteriorly with respect to the medial tibial plateau line, the cases were classified into group A (Figure 1A), while when the osteotomy line was inclined posteriorly with respect to the medial tibial plateau line, the cases were classified into group P (Figure 1B). Group A and group P consisted of 58 and 22 patients, respectively.

### 2.2. Operative Procedure

The preoperative plan was made with a double-limb standing antero-posterior radiograph of the full-length lower extremity. The Miniaci method was used to obtain the valgus correction target angle [21]. The realigned weight-bearing line was aimed at passing the Fujisawa point (62.5% of the tibial plateau from the medial edge) from the center of the femoral head [22]. Arthroscopy was performed before the osteotomy. Thereafter, an oblique skin incision was made on the anteromedial aspect of the proximal tibia, and the superficial medial collateral ligament was released. Two Kirschner wires were inserted from the upper border of the pes anserinus to the fibular head under the guidance of an image intensifier. Primary transverse osteotomy was conducted along these two Kirschner wires, and a secondary ascending osteotomy was performed posterior to the tibial tuberosity. After gradually opening the osteotomy site with four chisels, it was opened with a bone spreader up to the planned correction angle. The ratio of the anterior gap to the posterior gap of the opening was maintained at approximately 2 to 3 [17,18,23]. A TomoFix plate (DePuy Synthes, West Chester, PA, USA) was fixed with locking screws to the proximal tibia to maintain the osteotomy gap.

### 2.3. Reconstruction of 3D Computed Tomography Model

A postoperative CT scan was taken on the day of the operation. CT evaluations were performed using the CT scanner Sensation 64 (Siemens Healthcare, Erlangen, Germany). The tube parameters were 120 kVp and 135~253 mAs. The acquisition matrix was 512 × 512 pixels. The scan field of view was 134~271 mm, and the slice thickness was 0.6~1 mm. A CT scan was done with the knee in full extension. Digital Imaging and Communications in Medicine (DICOM) data were downloaded from the picture archiving and communication system (Centricity PACS, GE Medical System Information Technologies, Milwaukee, Wisconsin). The axial, coronal, and sagittal DICOM images were then imported into Mimics software (version 17; Materialise, Leuven, Belgium), and a 3D bone model of the femur and tibia treated with MOWHTO was reconstructed. The plate and locking screws were removed digitally from the 3D model, leaving only the tibia after osteotomy.

### 2.4. Measurement of Variables on 3D Reconstructed Model

**Figure 2:**
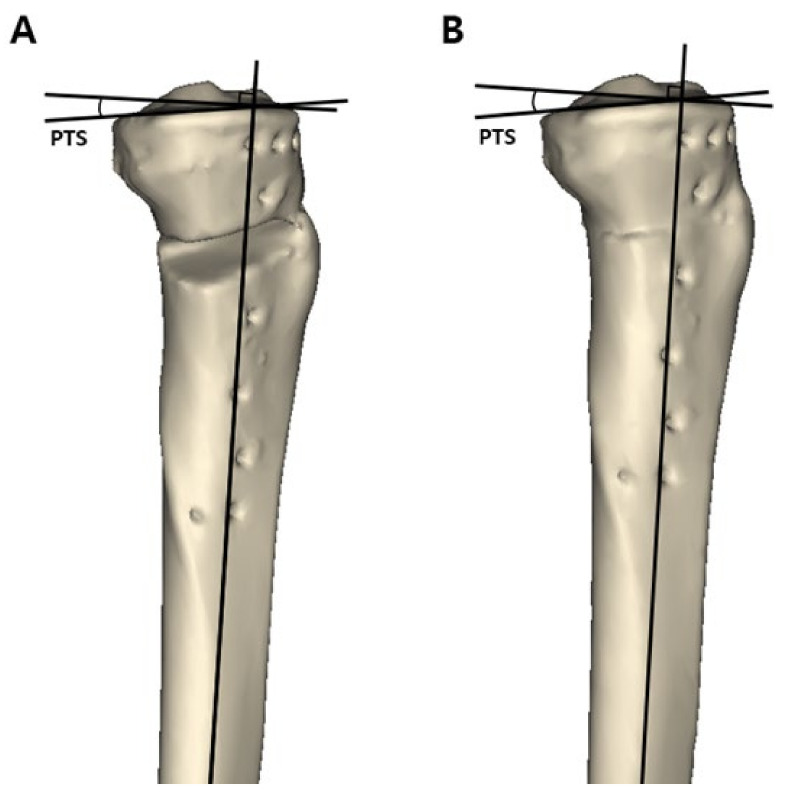
Measurement of the posterior tibial slope in a three-dimensional reconstructed model. The line perpendicular to the bisecting line of the tibial shaft and medial tibial plateau line was drawn on a true lateral view. The angle formed by these two lines was defined as the posterior tibial slope. (**A**) Postoperative posterior tibial slope. (**B**) Preoperative posterior tibial slope measured in the restored original preoperative tibia model. posterior tibial slope = posterior tibial slope.

**Figure 3:**
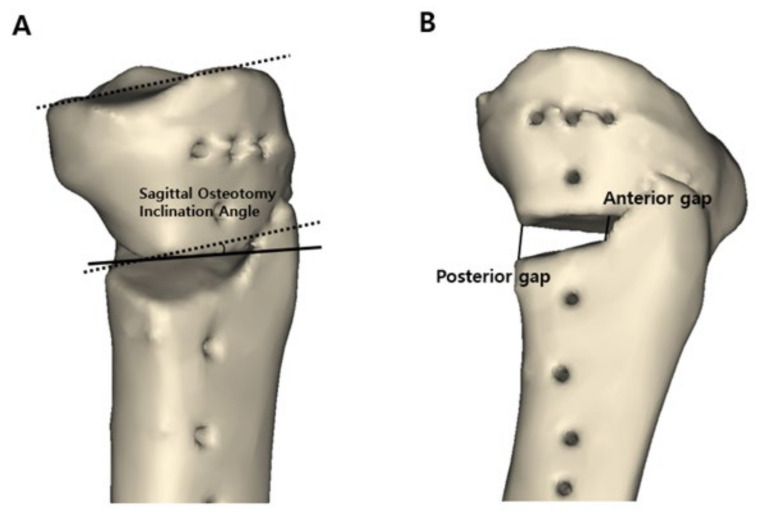
Measurement of the sagittal osteotomy inclination angle and anterior and posterior opening gap in a three-dimensional reconstructed model. (**A**) The sagittal osteotomy inclination angle was measured as the angle formed by the medial tibial plateau line (black dotted line) and osteotomy line on the anteromedial aspect of the lowermost part of the proximal tibial segment (black solid line) on the true lateral view. (**B**) Distances of anterior opening gap and posterior opening gap were measured in the three-dimensional reconstructed model. The anterior opening gap was measured at the medial edge of the frontal plane osteotomy site. The posterior opening gap was measured at the most prominent posteromedial edge of tibia.

Three major variables were measured in the 3D reconstructed model: (1) the preoperative and postoperative posterior tibial slope, (2) osteotomy inclination angle relative to the medial tibial plateau line in the sagittal plane, and (3) anterior and posterior opening gap and the ratio of these two osteotomy gaps. To measure the posterior tibial slope and osteotomy inclination angle in the sagittal plane, the 3D reconstructed model was aligned in a true lateral position. The true lateral position of the tibia was obtained by manipulating the femoral lateral and medial condyles to be superimposed on the basis of the method presented in a previous study [24]. When measuring variables, the 3D model of the femur was made invisible for convenience. After the true lateral view was completed, this view was captured because these two lines could not be located on the same plane. The posterior tibial slope was measured on the captured 3D true lateral view as previously described [25,26]. The line perpendicular to the bisecting line of the tibial shaft and the medial tibial plateau line were drawn. The angle formed by these two lines was defined as the posterior tibial slope (Figure 2A). To measure the preoperative original posterior tibial slope, the original tibia model was obtained by removing the osteotomy gap of the proximal tibia. The proximal and distal parts of the tibia were combined to the preoperative original tibia by rotating the proximal segment using the lateral cortex as a hinge. The original posterior tibial slope was measured in the same way (Figure 2B). To measure the osteotomy inclination angle in the sagittal plane, a medial tibial plateau line and a sagittal osteotomy line on the anteromedial aspect of the lowermost part of the proximal tibia segment were drawn on the captured 3D true lateral view. The angle formed by these two lines was defined as the osteotomy inclination angle (Figure 3A) [19]. When the front of the osteotomy line was inclined downward with respect to the line parallel to the medial tibial plateau, it was classified as positive, and if it was inclined upward, it was classified as negative. The osteotomy gap ratio was defined as the ratio between the distances of the anterior opening gap and posterior opening gap in the 3D reconstructed model. The anterior opening gap was measured at the medial edge of the frontal plane osteotomy site. The posterior opening gap was measured at the most prominent posteromedial edge of the tibia (Figure 3B) [17,27]. The osteotomy gap ratio (%) was calculated by dividing the anterior opening gap by the posterior opening gap and multiplying by 100. Measurements of variables were conducted by two independent orthopedic surgeons who did not participate in the MOWHTO and were blinded to the patients’ information to increase their reliability. The mean of the two numerical values was used.

### 2.5. Measurement on Plain Radiographs

**Figure 4:**
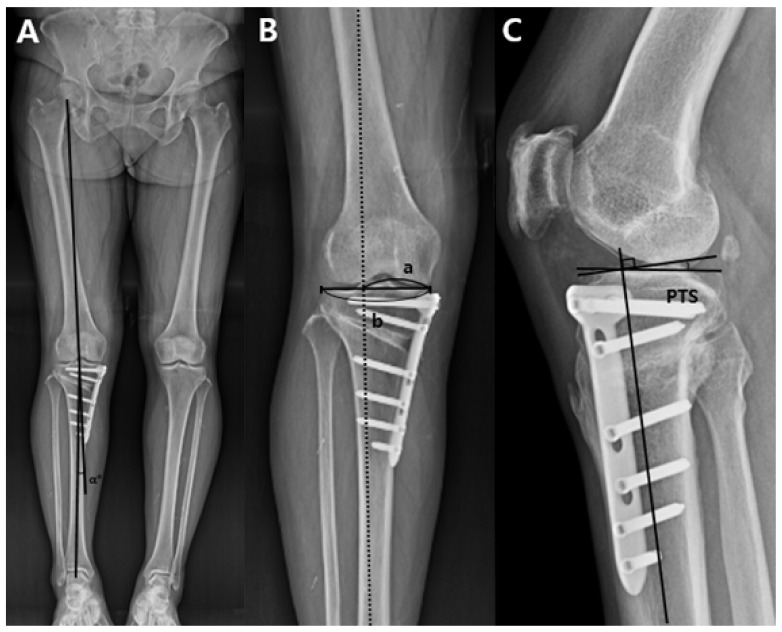
Measurement of the hip-knee-ankle angle, weight-bearing line ratio, and posterior tibial slope on the plain radiographs. (**A**) The hip-knee-ankle angle (α°) is formed by the first line drawn from the center of the femoral center to center of the tibial spine and the second line drawn from the center of the tibial spine of knee joint to the center of the superior articular surface of the talus in the ankle joint. The angle made by the intersection of these two lines was defined as the hip-knee-ankle angle. (**B**) The weight-bearing line was drawn from the center of the femoral head to the middle point of the superior articular surface of the talus on the full-length lower extremity standing radiograph. A tibial plateau line from the medial edge to the lateral edge of the proximal tibia on the joint surface was drawn thereafter. Weight-bearing line ratio (a/b) was calculated as the ratio of the distance from the medial edge of the proximal tibial plateau to the intersection of the weight-bearing line and the proximal tibial plateau line (a) to the entire length of the proximal tibial plateau line (b). The medial tibial edge was 0%, and the lateral tibial edge was 100%. (**C**) The posterior tibial slope is formed by the perpendicular line to the tibial shaft axis and the medial tibial plateau line on the true lateral view. posterior tibial slope = posterior tibial slope.

In addition to the measurement of variables in the 3D reconstructed model, the preoperative and postoperative hip–knee–ankle angle, weight-bearing line ratio, and posterior tibial slope were also measured using plain radiographs. Plain knee radiographs of the anteroposterior and lateral views were taken preoperatively and postoperatively on the day of the operation. Full-length lower extremity standing radiographs were also taken preoperatively and 6 months postoperatively. The hip-knee-ankle angle was measured on the full-length lower extremity standing radiograph with the first line from the femoral head center to the tibial spine center and the second line from the tibial spine center to the center of the superior articular surface of the talus. The angle made by the intersection of these two lines was defined as the hip–knee–ankle angle (Figure 4A) [28]. The weight-bearing line ratio was measured with the weight-bearing line and tibial plateau line. The weight-bearing line was drawn from the center of the femoral head to the middle point of the superior articular surface of the talus on the full-length lower extremity standing radiograph. A tibial plateau line from the medial edge to the lateral edge of the proximal tibia on the joint surface was drawn thereafter. The weight-bearing line ratio was calculated as the ratio of the distance from the medial edge of the proximal tibial plateau to the intersection of the weight-bearing line and the distance from the proximal tibial plateau line to the entire length of the proximal tibial plateau line. The medial tibial edge was 0%, and the lateral tibial edge was 100% (Figure 4B) [28]. Posterior tibial slope was also measured on the lateral view of the plain radiograph taken in a true lateral position [29]. The dots were marked 7 cm and 12 cm below the joint line on the anterior and posterior cortex of the tibia. Two lines connecting the two dots marked at 7 cm and 12 cm were drawn, and a line passing through the midpoint of these two lines was defined as the tibial shaft axis. The posterior tibial slope was measured as the angle formed by the line perpendicular to the tibial shaft axis and medial tibial plateau line (Figure 4C). These variables were also measured by two independent orthopedic surgeons. Measurements on the plain radiographs were made without knowing the measured values in the 3D model for each patient. The mean of the two numerical values was used.

### 2.6. Statistical Analysis

The normality of distribution test was assessed using the Kolmogorov–Smirnov test. After confirming that the data followed a normal distribution, an independent t-test was employed for continuous variables. A paired t-test was used to compare preoperative and postoperative variables. For categorical variables, the chi-square or Fisher’s exact test was used. Pearson correlation analysis was used to evaluate the correlation between variables (inclination angle, gap ratio, and correction angle) and the change in posterior tibial slope. Subsequently, multiple regression analysis was conducted to estimate the effect of the independent variable (inclination angle, gap ratio, and correction angle) on the dependent variable (change in posterior tibial slope). The intraclass correlation coefficient was calculated to assess the interobserver reliabilities of each measurement. The level of significance was set at *p* < 0.05. Statistical analyses were conducted using IBM SPSS Statistics for Windows software program (version 25.0; IBM, Armonk, NY, USA). The statistical power was calculated using G*Power (v 3.1) [30].

## 3. Results

### 3.1. Subjects

The mean age at the time of surgery was 55.6 years in group A and 56.1 years in group P. There were 14 male and 44 female patients in group A and eight male and 14 female patients in group P. There was no statistically significant difference between the groups regarding age at the time of surgery, sex, affected side, and body mass index (*p* > 0.05) (Table 1). 

### 3.2. Measurement with Plain Radiographs

On radiologic evaluation with plain radiographs, the preoperative values of hip-knee-ankle angle (group A = −7.2 ± 2.3°, group P = −7.8 ± 2.8°, *p* = 0.319), weight-bearing line ratio (group A = 19.3 ± 8.5%, group P = 18.0 ± 10.6%, *p* = 0.343), and posterior tibial slope (group A = 9.5 ± 3.1°, group P = 8.3 ± 2.7°, *p* = 0.117) did not differ significantly between the groups. The postoperative values of hip-knee-ankle angle (group A = 3.2 ± 2.3°, group P = 3.9 ± 2.3°, *p* = 0.772) and weight-bearing line ratio (group A = 61.1 ± 6.7%, group P = 63.9 ± 7.2%, *p* = 0.716) did not differ significantly between the groups. The correction angle did not differ significantly between the groups: group A = 11.0 ± 2.5°; group P = 11.6 ± 2.6° (*p* = 0.396). However, the postoperative posterior tibial slope (group A = 10.9 ± 4.0°, group P = 7.6 ± 3.2°, *p* = 0.001) and change in posterior tibial slope between the preoperative and postoperative values (group A = 1.4 ± 2.6°, group P = −0.8 ± 2.3°, *p* = 0.001) differed significantly between the groups (Table 2). A significant difference was also found in the comparison of posterior tibial slope before and after surgery for all patients (preoperative value = 9.1 ± 3.1°, postoperative value = 9.8 ± 4.1°, *p* = 0.008). The preoperative and postoperative values within each group were significantly different only in group A (group A: preoperative value = 9.5 ± 3.1°, postoperative value = 10.9 ± 4.0°, *p* < 0.001; group P: preoperative value = 8.3 ± 2.7°, postoperative value = 7.6 ± 3.2°, *p* = 0.137) (Table 3). The intraclass correlation coefficients for interobserver reliability on plain radiograph were 0.748 (95% confidence interval (CI), 0.607~0.838) for the preoperative posterior tibial slope and 0.759 (95% CI, 0.625~0.846) for the postoperative posterior tibial slope. The intraclass correlation coefficients for hip-knee-ankle angle and weight-bearing line ratio are shown in Appendix A.

### 3.3. Measurement with the 3D Reconstructed Model

In the 3D reconstructed model, the posterior tibial slope, osteotomy inclination angle relative to the medial tibial plateau line in the sagittal plane, and the ratio between the anterior and posterior osteotomy opening gaps were measured. There was no significant difference in the preoperative posterior tibial slope between the groups (group A = 9.7 ± 2.9°, group P = 8.7 ± 2.7°, *p* = 0.143). However, the postoperative posterior tibial slope (group A = 10.7 ± 3.0°, group P = 7.7 ± 2.7°, *p* < 0.001) and change in posterior tibial slope before and after surgery (group A = 1.0 ± 0.8°, group P = −0.9 ± 0.8°, *p* < 0.001) differed significantly between the groups (Table 4). A significant difference was also found in the comparison of the posterior tibial slope measured with the 3D reconstructed model before and after surgery for all patients (preoperative value = 9.4 ± 2.9°, postoperative value = 9.9 ± 3.2°, *p* = 0.001). When comparing the preoperative and postoperative values within each group, there were significant differences in both groups A and P (group A: preoperative value = 9.7 ± 2.9°, postoperative value = 10.7 ± 3.0°, *p* < 0.001; group P: preoperative value = 8.7 ± 2.7°, postoperative value = 7.7 ± 2.7°, *p* < 0.001) (Table 3). The intraclass correlation coefficients for interobserver reliability using the 3D reconstructed model were 0.928 (95% CI, 0.888~0.954) for the preoperative posterior tibial slope and 0.937 (95% CI, 0.902~0.960) for the postoperative posterior tibial slope. The mean osteotomy inclination angle in the sagittal plane was 4.9 ± 3.2 (range, 0.5° to 15.0°) in group A, and −3.1 ± 2.5 (range, −0.2° to −10.6°) in group P (*p* < 0.001). The anterior opening gap was 8.2 ± 2.2 mm (range, 4.2–14.5), the posterior opening gap was 11.8 ± 2.6 mm (range, 7.2–19.6), and the gap ratio was 69.7 ± 8.4% in group A. The anterior opening gap was 8.3 ± 1.4 mm (range, 5.4–10.4), the posterior opening gap was 12.6 ± 2.4 mm (range, 8.1–17.0), and the gap ratio was 66.3 ± 9.5% in group P. The gap ratio did not differ significantly between the groups (*p* = 0.118) (Table 4). The intraclass correlation coefficients for the osteotomy inclination angle in the sagittal plane and the osteotomy opening gaps are shown in appendix 1. The statistical power assessed with G*Power [30] was 99.2% regarding the postoperative posterior tibial slope.

**Figure 5:**
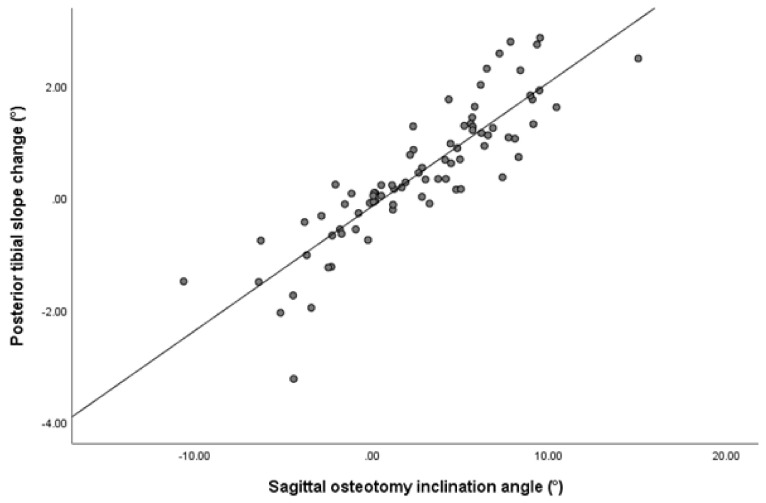
Scatter plot of Pearson correlation analysis showing the correlation between sagittal osteotomy inclination angle and the posterior tibial slope change. The sagittal osteotomy inclination angle has a significantly positive correlation with the posterior tibial slope change (r = 0.875, *p* < 0.001).

According to the Pearson correlation analysis, there was a significant correlation between two parameters, namely osteotomy inclination angle in sagittal plane and opening gap ratio and posterior tibial slope change. The Pearson correlation coefficient (r) was 0.875 (*p* < 0.001) for the osteotomy inclination angle (Figure 5) and 0.233 (*p* = 0.038) for the gap ratio (Table 5). According to the subsequent multivariate regression analysis to identify significant independent variables affecting posterior tibial slope change, the only significant factor among the variables was the osteotomy inclination angle in the sagittal plane (β coefficient = 0.216, *p* < 0.001) (Table 6).

## 4. Discussion

The posterior tibial slope changes after MOWHTO, but little is known about the effect of the osteotomy inclination angle in the sagittal plane on the change in the posterior tibial slope. To investigate this effect, changes in the posterior tibial slope before and after surgery were measured, and the tendency and extent of the change in the posterior tibial slope were assessed by comparing two groups: anterior and posterior osteotomy inclination in the sagittal plane. According to the principal findings of this study, although the mean gap ratio between the anterior and posterior openings was maintained at an appropriate value, approximately 2:3, as recommended in a previous study [17], there was a significant difference in the posterior tibial slope before and after MOWHTO. In addition, the osteotomy inclination angle in the sagittal plane had a significant effect on the change in the posterior tibial slope. The anterior osteotomy inclination angle with the forward part of the osteotomy plane heading downward with respect to the medial tibial plateau line led to an increase in the postoperative posterior tibial slope. In contrast, the posterior osteotomy inclination angle led to a decrease in the postoperative posterior tibial slope. 

Previous studies have noted that the posterior tibial slope changes after MOWHTO [12,18]. A recent meta-analysis also reported that posterior tibial slope increased by 2.02° after MOWTHO [16]. Maintaining the posterior tibial slope before and after surgery is important to avoid unpredictable adverse effects. An increase in the posterior tibial slope could cause anterior translation of the tibia and increase the load on the anterior cruciate ligament [11,12]. An increased posterior tibial slope could also cause a redistribution of pressure into the posterior tibia in the anterior cruciate ligament-deficient knee, causing degenerative changes in articular cartilage [13] and an increase of vertical vector force onto the patellofemoral joint, which could lead to osteoarthritis of the patellofemoral joint [14,15]. In addition to the knee joint, it has been reported that the tibial slope also has important biomechanical implications on the ankle joint [31]. For these reasons, it is important to maintain the posterior tibial slope after MOWHTO, but there are practical difficulties in maintaining the posterior tibial slope during surgery because the proximal tibia is triangular, and osteotomy is performed from the anteromedial aspect without seeing the overall shape of the tibia. To avoid changes in the posterior tibial slope after MOWHTO, maintaining a constant ratio of the osteotomy opening gap between the anterior and posterior cortices has been emphasized [17,18]. Noyes et al. [18] reported that to maintain the original posterior tibial slope, the anterior gap should be one half of the posterior gap, and Song et al. [17] demonstrated that the normal posterior tibial slope can be unchanged if the anterior opening gap is approximately 67% of the posterior opening gap. To maintain the opening gap ratio, various factors leading to postoperative changes in the posterior tibial slope should be considered during surgery, such as incomplete posterior osteotomy, posterolateral hinge position, and an anteriorly placed plate affecting the gap ratio between the anterior and posterior cortices [26,32,33,34]. However, the results of this study showed that, although the mean opening gap ratio was maintained at an appropriate value, approximately 2:3 [17], the posterior tibial slope could change after MOWHTO. According to the comparison of posterior tibial slopes before and after surgery measured with 3D reconstructed models for all patients, there was a significant increase in the posterior tibial slope after surgery (preoperative value = 9.4 ± 2.9°, postoperative value = 9.9 ± 3.2°, *p* = 0.001). Consequently, it is necessary to identify additional factors affecting the change in the posterior tibial slope after MOWHTO. 

According to the results of this study, another factor affecting the change in the posterior tibial slope was the inclination angle of osteotomy in the sagittal plane. Few studies have dealt with the osteotomy inclination angle in relation to the posterior tibial slope [19,35]. A previous study recommended an osteotomy line parallel to the joint line to avoid inadvertent alteration of the posterior tibial slope [35]. Lee et al. [19] investigated the inclination angle of osteotomy in the sagittal plane and noted that only 12.9% of cases were conducted parallel to the medial joint line and 87.1% of osteotomy lines were anteriorly inclined. Anterior inclination of osteotomy in the sagittal plane was reported to increase the posterior tibial slope. In addition to these previous studies, our study comprehensively assessed how anterior or posterior inclination of the sagittal osteotomy line affects the change in the posterior tibial slope after MOWHTO, and the correlation between the osteotomy inclination angle and postoperative posterior tibial slope angle. In our study, 72.5% of patients had anteriorly inclined osteotomy, while 27.5% of patients had posteriorly inclined osteotomy. Similar to a previous study [19], the majority of osteotomy lines in the sagittal plane were anteriorly inclined. According to the data measured with 3D reconstructed models, the preoperative posterior tibial slope was 9.7 ± 2.9° and postoperative posterior tibial slope was 10.7 ± 3.0° in group A. The preoperative posterior tibial slope was 8.7 ± 2.7° and the postoperative posterior tibial slope was 7.7 ± 2.7° in group P. The mean osteotomy inclination angle in the sagittal plane was 4.9 ± 3.2 in group A and −3.1 ± 2.5 in group P. Although there was no significant difference in the osteotomy opening gap ratio between the groups, the postoperative posterior tibial slope changed in different direction depending on the difference in the inclination angle in sagittal plane. When the osteotomy line was inclined anteriorly with respect to the medial tibial plateau line, the postoperative posterior tibial slope tended to increase. In contrast, when the osteotomy line was inclined posteriorly, the postoperative posterior tibial slope tended to decrease. Both the Pearson correlation (r = 0.875, *p* < 0.001) and multiple regression analyses (β coefficient = 0.216, *p* < 0.001) also showed a significant association between the osteotomy inclination angle in the sagittal plane and change in posterior tibial slope. Accordingly, to avoid inadvertent effects on the kinematics of the knee joint, close attention needs to be paid to maintaining the sagittal osteotomy line parallel to the medial joint line during MOWHTO. In addition, in cases where the treatment effect is expected by intentionally adjusting the posterior tibial slope, such as for osteoarthritis with anteroposterior instability, the posterior tibial slope could be controlled by varying the inclination angle of the osteotomy in the sagittal plane [12].

Another notable finding in this study was that measurements with 3D reconstructed models were more reliable than measurement with plain radiographs. When measured with plain radiographs, the intraclass correlation coefficients for interobserver reliability were 0.748 (95% CI, 0.607~0.838) for the preoperative posterior tibial slope and 0.759 (95% CI, 0.625~0.846) for the postoperative posterior tibial slope. However, when measured with 3D reconstructed models, the intraclass correlation coefficients for interobserver reliability were 0.928 (95% CI, 0.888~0.954) for the preoperative posterior tibial slope and 0.937 (95% CI, 0.902~0.960) for the postoperative posterior tibial slope. In comparing the preoperative and postoperative posterior tibial slopes in group P, measured values with plain radiographs did not differ significantly, but measured values with 3D reconstructed models differed significantly. There is still no established method for measuring the posterior tibial slope. A more reliable and generally agreeable method uses the medial tibial plateau line and anatomical axis of the proximal tibial shaft or tibial cortex line [36]. However, the measurement method using plain radiographs has disadvantages such as poor reproducibility due to the difficulties in obtaining true lateral images of the tibia caused by rotation and distinguishing between the medial and lateral tibial plateau because they overlap [37]. For these reasons, CT scans and magnetic resonance imaging have been recently used for measurement of the posterior tibial slope. In this study, measurements were performed with 3D reconstructed models using the method presented in previous studies [25,26]. The results of our study also demonstrated that measurements with 3D reconstructed models obtain more consistent values with higher interobserver reliability.

This study had several limitations. Firstly, this study was based on retrospectively collected data. In order to obtain a more solid conclusion, a prospective study design by means of a cadaver study or finite elements is needed. Secondly, a preoperative CT scan was not performed. The preoperative 3D tibia model was reconstructed by digitally removing the osteotomy gap from the postoperative 3D tibia model using a lateral hinge as a rotation axis. Although the preoperative 3D tibia model was restored as close to the actual preoperative tibia as possible, there could be a difference with the actual preoperative tibia. Thirdly, the number of patients in each study group was different. The majority of osteotomies in the sagittal plane were anteriorly inclined. Therefore, the number of patients in group A was larger than in group P. The relatively small number of patients could decrease statistical power. However, the calculated statistical power was 99.2%. Thus, the small number of patients in group P was not considered a serious problem. Fourthly, there was a statistically significant difference between the preoperative and postoperative posterior tibial slopes in all patients and in each group. There was also a statistically significant difference in postoperative posterior tibial slopes between the groups. However, as with the minimal clinically important difference, it has not been revealed how much the difference in posterior tibial slope actually affects the clinical outcomes. Accordingly, to determine the clinical effect of the change in posterior tibial slope on functional outcomes, a more in-depth study with long-term clinical follow-up results is needed.

## 5. Conclusions

Although the osteotomy gap ratio between the anterior and posterior gaps was kept at an appropriate value, the posterior tibial slope changed after MOWHTO according to the osteotomy inclination angle in the sagittal plane. The postoperative posterior tibial slope tended to increase when the osteotomy line was inclined anteriorly with respect to the medial tibial plateau line but decreased when the osteotomy line was inclined posteriorly. To avoid inadvertent effects on the kinematics of the knee joint, close attention needs to be paid to maintaining the sagittal osteotomy line parallel to the medial joint line during MOWHTO. This study would be helpful to understanding the effect of the osteotomy inclination angle in the sagittal plane on the change in the posterior tibial slope after MOWHTO.

## Figures and Tables

**Table 1:** Patient’s demographic data.

Variable	Group A (*n* = 58)	Group P (*n* = 22)	*p*-Value
Age * (years)	55.6 ± 5.3	56.1 ± 3.4	0.648
Sex †			0.274
Male	14	8	
Female	44	14	
Affected side †			0.617
Right	28	12	
Left	30	10	
Body mass index * (kg/m^2^)	27.1 ± 3.5	27.0 ± 2.7	0.845

* Values are given as the mean ± standard deviation.; † Values are given as *n*.

**Table 2:** Preoperative and postoperative measurement on the plain radiograph.

Variable	Group A (*n* = 58)	Group P (*n* = 22)	*p*-Value
Hip-knee-ankle angle (°)			
Preoperative value	−7.2 ± 2.3	−7.8 ± 2.8	0.319
Postoperative value	3.2 ± 2.3	3.9 ± 2.3	0.772
Weight-bearing line ratio (%)			
Preoperative value	19.3 ± 8.5	18.0 ± 10.6	0.343
Postoperative value	61.1 ± 6.7	63.9 ± 7.2	0.716
Correction angle (°)	11.0 ± 2.5	11.6 ± 2.6	0.396
Posterior tibial slope (°)			
Preoperative value	9.5 ± 3.1	8.3 ± 2.7	0.117
Postoperative value	10.9 ± 4.0	7.6 ± 3.2	0.001
Posterior tibial slope change (°)	1.4 ± 2.6	−0.8 ± 2.3	0.001

Values are given as the mean ± standard deviation.

**Table 3:** Comparison between preoperative and postoperative measurement of posterior tibial slope on the plain radiograph and three-dimensional model.

Variable	Preoperative PTS	Postoperative PTS	*p*-Value
Plain radiography			
All patients (°)	9.1 ± 3.1	9.8 ± 4.1	0.008
Group A (°)	9.5 ± 3.1	10.9 ± 4.0	<0.001
Group P (°)	8.3 ± 2.7	7.6 ± 3.2	0.137
Three-dimensional model			
All patients (°)	9.4 ± 2.9	9.9 ± 3.2	0.001
Group A (°)	9.7 ± 2.9	10.7 ± 3.0	<0.001
Group P (°)	8.7 ± 2.7	7.7 ± 2.7	<0.001

Values are given as the mean ± standard deviation. PTS = Posterior tibial slope.

**Table 4:** Preoperative and postoperative measurement in the three-dimensional model.

Variable	Group A (*n* = 58)	Group P (*n* = 22)	*p*-Value
Posterior tibial slope (°)			
Preoperative value	9.7 ± 2.9	8.7 ± 2.7	0.143
Postoperative value	10.7 ± 3.0	7.7 ± 2.7	<0.001
Posterior tibial slope change (°)	1.0 ± 0.8	−0.9 ± 0.8	<0.001
Inclination angle (°)	4.9 ± 3.2	−3.1 ± 2.5	<0.001
Gap ratio (%)	69.7 ± 8.4	66.3 ± 9.5	0.118
Anterior opening gap (mm)	8.2 ± 2.2	8.3 ± 1.4	0.916
Posterior opening gap (mm)	11.8 ± 2.6	12.6 ± 2.4	0.185

Values are given as the mean ± standard deviation.

**Table 5:** Correlation between variables and change in posterior tibial slope.

Variable	Posterior Tibial Slope Change	
	Pearson Correlation Coefficient (r)	*p* Value
Inclination angle	0.875	<0.001
Gap ratio	0.233	0.038
Correction angle	−0.170	0.133

**Table 6:** Multivariate regression analysis for change in posterior tibial slope.

Variable	β Coefficient	Standard Error	*p* Value
Inclination angle	0.216	0.014	<0.001
Gap ratio	0.011	0.008	0.149
Correction angle	−0.026	0.027	0.332

## Data Availability

The data presented in this study may be available on request from the corresponding author. The data are not publicly available due to privacy and ethical considerations.

## Appendix A

**Table A1:** Intraclass correlation coefficient.

Variable	ICC	95% Confidence Interval
Hip-Knee-Ankle angle		
Preoperative	0.907	0.868–0.936
Postoperative	0.916	0.881–0.943
Weight-bearing line ratio		
Preoperative	0.923	0.892–0.948
Postoperative	0.927	0.896–0.950
Osteotomy inclination angle	0.942	0.918–0.960
Osteotomy opening gap		
Anterior opening gap	0.936	0.910–0.956
Posterior opening gap	0.930	0.902–0.952

ICC = Intraclass correlation coefficient.
